# Effect of MiR-210 on the Chemosensitivity of Breast Cancer by Regulating JAK-STAT Signaling Pathway

**DOI:** 10.1155/2021/7703159

**Published:** 2021-08-11

**Authors:** Zeyu Xing, Xin Wang, Jiaqi Liu, Gang Liu, Menglu Zhang, Kexin Feng, Xiang Wang

**Affiliations:** Department of Breast Cancer, National Cancer Center/National Clinical Research Center for Cancer/ Cancer Hospital, Chinese Academy of Medical Sciences and Peking Union Medical College, Beijing 10021, China

## Abstract

The study is aimed at exploring the effect of microribonucleic acid- (miR-) 210 on the chemosensitivity of breast cancer and its potential molecular mechanism. Cell Counting Kit-8 (CCK-8) was applied to detect the half maximal inhibitory concentration (IC_50_) of cisplatin (DDP) on cell, and quantitative polymerase chain reaction (qPCR) was carried out to measure the relative expression level of miR-210. The IC_50_ value of DDP on cells was detected via CCK-8 after downregulating the expression of miR-210 in MCF-7/DDP cells. Flow cytometry and terminal deoxynucleotidyl transferase dUTP nick end labeling (TUNEL) confirmed the effect of themiR-210 downregulation on the apoptosis of drug-resistant MCF-7/DDP cells. Besides, the impacts of the miR-210 downregulation on apoptosis-related proteins and Janus-activated kinase- (JAK-) signal transducer and activator of transcription (STAT) signaling pathway-related proteins were examined by Western blotting. The interaction between miR-210 and the target protein was detected through luciferase activity assay, qPCR, and Western blotting. Drug-resistant MCF-7/DDP cells had significantly stronger resistance to DDP and a remarkably higher expression level of miR-210 than control parental MCF-7 cells (*p* < 0.05). After the downregulation of the miR-210 expression, MCF-7/DDP cells had markedly reduced resistance but obviously increased sensitivity to DDP (*p* < 0.05). MiR-210 downregulation increased the apoptosis of MCF-7/DDP cells (*p* < 0.05). In addition, after miR-210 was knocked down, the expression level of b-cell lymphoma 2 (Bcl-2) was decreased, while the expression levels of Bcl-2-associated X protein (Bax) and cysteinyl aspartate-specific proteinase-3 (caspase-3) were increased. Besides, miR-210 was able to suppress the expression of protein inhibitor of the activated STAT 4 (PIAS4) gene by directly targeting its 3′ untranslated region (3′UTR). The expression of miR-210 has a correlation with chemoresistance of breast cancer MCF-7 cells. MiR-210 regulates the JAK-STAT signal transduction pathway by targeting PIAS4, thus exerting an effect on breast cancer chemosensitivity.

## 1. Introduction

Breast cancer (BC) is the second most common cancer worldwide and is also the fifth major cause of cancer-related deaths among women [1]. A recent report shows that breast cancer ranks first among young women aged 20-39 years old in terms of the number of new cases and death toll around the world [2]. In 2017, about 252,710 women are expected to be newly diagnosed with breast cancer in the United States, and about 40,610 women will die of the disease [3]. Importantly, breast cancer is not a local tumor, but it involves lymph node or other organ metastasis leading to death. Therefore, the prediction, prevention, and treatment of breast cancer metastasis are of great importance to increase the survival rate of breast cancer patients [4, 5].

Currently, comprehensive treatment based on surgery and chemotherapy is the main clinical treatment strategy for breast cancer. However, chemotherapeutic drugs may kill cancer cells and damage normal tissues and cells at the same time, which easily triggers a series of complications seriously affecting the quality of life of patients. Chemotherapeutic drugs for breast cancer are still based on cisplatin (DDP) drugs. Despite the successful application of these drugs, the insensitivity of cancer to chemotherapeutic drugs in clinical application greatly reduces the efficacy of drugs and brings irresistible resistance to the treatment of patients' diseases [6, 7]. Drug resistance caused by long-term chemotherapy severely limits the clinical efficacy and outcomes of breast cancer treatment. Therefore, exploring natural and low toxic drugs that can specifically restore the sensitivity of breast cancer cells to chemotherapeutic drugs and clarifying the detailed mechanism of chemical sensitization are of vital significance, which have become urgent and challenging problems globally.

Microribonucleic acids (miRNAs) are a kind of noncoding RNAs with a length of about 22 nucleotides. They negatively regulate the posttranscriptional gene expression by inhibiting targeted messenger RNA (mRNA) translation or by partially binding the 3′ untranslated region (UTR) of mRNA [8]. At present, it is widely believed that miRNAs can regulate various cellular processes in normal and tumor cells. Studies have also revealed that abnormally expressed miRNAs act as tumor suppressors or activators to participate in the occurrence and metastasis of breast tumors [9, 10]. Zhang et al. [11] studied the function of resveratrol in chemosensitivity of adriamycin in low-invasive breast cancer and determined the targeted miRNAs of resveratrol and its key target proteins related to cell activity. Resveratrol was discovered to be a potential inducer enhancing the chemosensitivity of breast cancer. MiR-122-5p participates in cell cycle arrest by targeting b-cell lymphoma 2 (Bcl-2) and CDK. A study of Yu et al. has demonstrated that miR-221 promotes the resistance of osteosarcoma cells to DDP by targeting PPP2R2A [12]. Liu Wenbin and Gao [13] have shown that miR-210 is expressed at a significantly high level in tumor tissues of breast cancer patients. And it is closely related to the patient's disease progression, tumor malignancy, and prognosis, and it is a potential molecular marker and targeted therapy site. However, the relationship between drug resistance and breast cancer remains unclear. In this research, therefore, the relationship between the miR-210 expression and chemoresistance of breast cancer MCF-7 cells was primarily investigated.

## 2. Materials and Methods

### 2.1. Materials

A human breast cancer cell line MCF-7 was purchased from the American Type Culture Collection, a human breast cancer cell line MCF-7 resistant to DDP (MCF-7/DDP) from Shanghai Cell Bank of the Chinese Academy of Sciences, DMEM high-glucose medium and fetal bovine serum (FBS) from Sijiqing Bioengineering Materials Co., Ltd. (Hangzhou), miRNA fluorescence quantitative detection kit from Tiangen Biotech Co., Ltd. (Beijing), TRIzol Reagent and Lipofectamine 3000 transfection reagent from Invitrogen (USA), miR-210 inhibitor and mimics from GenePharma (Shanghai), apoptosis detection kit from Dojindo Molecular Technologies Inc. (Shanghai), pmirGLO plasmid and dual-luciferase detection system from Promega (USA), in situ cell apoptosis detection kit from Sigma (USA), antibodies against Bcl-2-associated X protein (Bax), Bcl-2, cysteinyl aspartate-specific proteinase 3 (Caspase-3), phosphorylated janus-activated kinase (p-JAK), JAK, phosphorylated signal transducer and activator of transcription (p-STAT), STAT, and protein inhibitor of activated STAT 4 (PIAS4) from Santa Cruz (USA), anti-*β*-actin antibody from Sigma (USA), real-time quantitative polymerase chain reaction (qPCR) instrument from Bio-Rad (USA), and a FACScan flow cytometer from BD (Switzerland).

### 2.2. Cell Culture and miRNA Transfection

Both human breast cancer cell line MCF-7 and drug-resistant cell line MCF-7/DDP were cultured in DMEM high-glucose medium containing 10% FBS and penicillin/streptomycin (100 mg/mL) and placed in a humidified incubator with 5% CO_2_ at 37°C. The cells were transfected using Lipofectamine 3000 transfection reagent according to the manufacturer's instructions.

### 2.3. Total RNA Extraction and Quantitative Reverse Transcription PCR (qRT-PCR)

Cells were lysed by TRIzol Reagent, and the total RNA was isolated. To determine the gene expression, 1 *μ*g of the total RNA was used to synthesize complementary deoxyribonucleic acids (cDNAs) with the total volume of 20 *μ*L, and 0.4 *μ*L of cDNA products was utilized for qPCR using a RT kit. miRNA qPCR was carried out with 0.5 *μ*L of cDNAs as a template using the miRNA fluorescence quantitative detection kit. Specific primers for miR-210 and endogenous reference U6 were synthesized by Beijing AuGCT biotechnology Co., Ltd. The relative expression of the target gene was calculated using the 2^-*ΔΔ*Cq^ method. Primers are shown below: miR-210: F: 5′-GTGCAGGGTCCGAGGT-3′ and R: 5′-CTGTGCGTGTGACAGCGGCTGA-3′, endogenous reference U6: F: 5′-CTCGCTTCGGCAGCACA-3′ and R: 5′-AACGCTTCACGAATTTGCGT-3′, PIAS4: F: 5′-GTGGGCCGGAGTAAGAGTG-3′ and R: 5′-TCAGGGCTACAGTCAAACTGC-3′, and endogenous reference GAPDH: F: 5′-CTCCTCCACCTTTGACGCTG-3′ and R: R′-TCCTCTTGTGCTCTTGCTGG-3′.

### 2.4. Detection of Cell Proliferation via Cell Counting Kit-8 (CCK-8)

Cell proliferation was determined using the CCK-8 kit on the basis of the manufacturer's instructions. Briefly, the cells were seeded in a 96-well plate at 2 × 10^3^ cells/well. After continuous culture in an incubator for 24, 48, and 72 h, 10 *μ*L of CCK-8 solution was added to each well for incubation at 37°C for another 2 h. Then, the absorbance of the mixed solution was measured at 450 nm using a microplate reader.

### 2.5. Detection of Cell Apoptosis Rate via Flow Cytometry

Cells were collected and washed with ice-cold PBS for 3 times. Subsequently, the cells were stained by Annexin V-FITC and PI using the cell cycle detection kit, and cell apoptosis was detected by flow cytometry.

### 2.6. Determination of the Number of Apoptotic Cells through Terminal Deoxynucleotidyl Transferase dUTP Nick End Labeling (TUNEL)

Cells were fixed and permeabilized. The number of apoptotic cells was determined by the TUNEL method using the in situ apoptosis detection kit. Then, the apoptotic cells were observed and photographed under a fluorescence microscope. Green fluorescence represented positive cells, while blue fluorescence represented nuclei. Finally, TUNEL-positive cells and total cells were analyzed by ImageJ software.

### 2.7. Detection via Western Blotting

The cultured cells were collected and added with RIPA buffer containing benzyl sulfonyl fluoride, protease inhibitor, and phosphatase inhibitor and lysed on ice for 30 min. After that, the lysate was centrifuged at 12,000 g and 4°C for 15 min. The proteins in the supernatant were collected, whose concentration was determined by the BCA assay. Thereafter, loading buffer was added, followed by boiling. Next, 40 *μ*g of the total proteins was taken and separated through 10% sodium dodecyl sulfate-polyacrylamide gel electrophoresis. After electrophoresis, the proteins were transferred onto a polyvinylidene fluoride membrane at 100 V. The membrane was sealed in 5% skim milk for 2 h and then incubated with primary antibody at 4°C overnight. Subsequently, the membrane was rinsed with TBST for three times and incubated with the secondary antibody at room temperature for 1 h, followed by washing with TBST for three times again. Ultimately, the target proteins were examined on the ECL color development system.

### 2.8. Detection of Luciferase Activity

Mutant (Mut) target sequences comprising a part of PIAS4 3′ untranslated region (3′-UTR) or miR-210 of the target sequence were cloned to the 3′ end of the luciferase coding sequence of pmirGLO to construct pmirGLO-PIAS4-wild type (WT) and pmirGLO-PIAS4-Mut. The constructs were confirmed by DNA sequencing. Then, the cells were seeded in a 24-well plate at about 2 × 10^5^ cells/well and cotransfected with 80 ng of pmirGLO-PIAS4-WT or -Mut and 8 pmol of mimics or negative control (NC), and the cells were collected 48 h later. The firefly and Ranilla luciferase activities were measured via the dual-luciferase report analysis system.

### 2.9. Statistical Methods

SPSS 21.0 software was adopted for the analysis of the data in this study. The data were expressed as mean ± standard deviation (^−^*x* ± s). ANOVA was employed for statistical analysis. *p* < 0.05 suggested that the difference was statistically significant.

## 3. Results

### 3.1. Relationship between the miR-210 Expression and Chemoresistance of Breast cancer MCF-7 Cells

**Figure 1:**
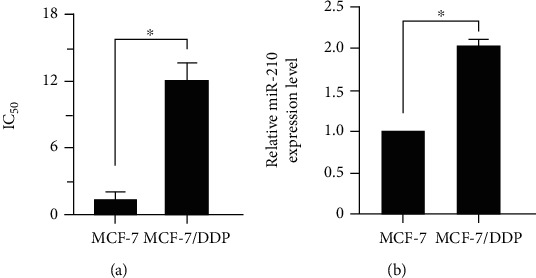
Relationship between the miR-210 expression and chemoresistance of breast cancer MCF-7 cells. (a) IC_50_ value of DDP on breast cancer drug-resistant cell line MCF-7/DDP and control parental cell line MCF-7 detected by CCK-8. (b) The relative expression level of miR-210 in breast cancer drug-resistant cell line MCF-7/DDP and control parental cell line MCF-7 examined via qRT-PCR (^∗^*p* < 0.05).

In order to explore the relationship between the miR-210 expression and chemoresistance of breast cancer MCF-7 cells, the half maximal inhibitory concentration (IC_50_) of DDP on breast cancer MCF-7/DDP cells and control parental MCF-7 cells was detected through CCK-8. The results (Figure 1(a)) revealed that the resistance of MCF-7/DDP cells to DDP was significantly stronger than that of MCF-7 cells (*p* < 0.05). The relative expression level of miR-210 in the two cell lines was measured via qPCR, and it was found that the expression level of miR-210 in MCF-7/DDP cells was remarkably higher than that in control parental MCF-7 cells (*p* < 0.05), indicating that the expression of miR-210 may be related to the chemoresistance of breast cancer MCF-7 cells.

### 3.2. Effect of the Downregulation of miR-210 on the Chemosensitivity of Drug-Resistant MCF-7/DDP Cells

**Figure 2:**
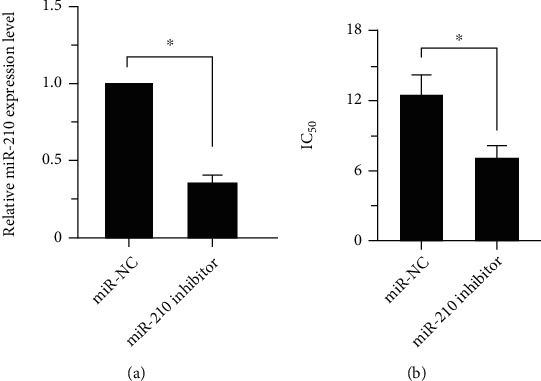
Effect of the downregulation of miR-210 on the chemosensitivity of drug-resistant MCF-7/DDP cells. (a) MiR-210 knock-down efficiency in MCF-7/DDR cells tested by qRT-PCR. (b) The IC_50_ value of DDP on cells detected by CCK-8 after downregulating the expression of miR-210 in MCF-7/DDP cells (^∗^*p* < 0.05).

In MCF-7/DDP cells, the knock-down efficiency of miR-210 after transfection with miR-210 inhibitor and miR-NC was tested by qRT-PCR. The results (Figure 2(a)) manifested that the expression level of miR-210 in MCF-7/DDP cells transfected with miR-210 inhibitor was evidently reduced compared with that in cells transfected with miR-210 NC (*p* < 0.05). After the downregulation of the miR-210 expression in MCF-7/DDP cells, CCK-8 was adopted to measure the IC_50_ value of DDP on cells. As shown in Figure 2(b), the resistance of MCF-7/DDP cells to DDP was remarkably reduced after downregulating the expression of miR-210, suggesting that the sensitivity to DDP is notably increased (*p* < 0.05).

### 3.3. Influence of the Downregulation of miR-210 on the Apoptosis of Drug-Resistant MCF-7/DDP Cells

**Figure 3:**
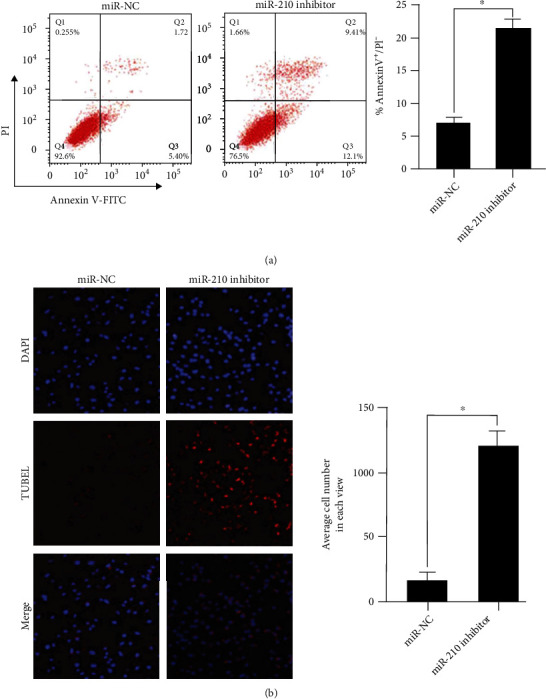
Influence of the downregulation of miR-210 on the apoptosis of drug-resistant MCF-7/DDP cells. (a) Influence of the miR-210 downregulation on the apoptosis rate of drug-resistant MCF-7/DDP cells determined by flow cytometry (^∗^*p* < 0.05). (b) Effect of the miR-210 downregulation on the apoptosis rate of drug-resistant MCF-7/DDP cells determined by the TUNEL assay (^∗^*p* < 0.05).

Flow cytometry was employed to determine the influence of the miR-210 downregulation on the apoptosis rate of drug-resistant MCF-7/DDP cells. According to the results (Figure 3), the apoptosis rate of cells in the miR-210 knock-down group was prominently raised in comparison with that in the control group (*p* < 0.05). It can be seen that the overexpression of miR-124 promotes cell apoptosis, implying that the downregulation of miR-210 can markedly facilitate the apoptosis of drug-resistant MCF-7/DDP cells. At the same time, the TUNEL assay was conducted to determine the effect of the miR-210 downregulation on the apoptosis rate of drug-resistant MCF-7/DDP cells. It was discovered that compared with control group, the knock-down of miR-210 obviously increased the number of apoptotic MCF-7/DDP cells (*p* < 0.05) (Figure 3(b)). Therefore, the above findings indicate that the miR-210 downregulation increases the apoptosis rate of MCF-7/DDP cells.

### 3.4. Impacts of the Downregulation of miR-210 on the Expressions of Apoptosis-Related Proteins in Drug-Resistant MCF-7/DDP Cells

**Figure 4:**
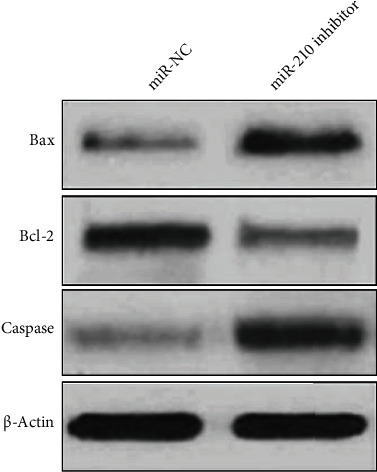
Impacts of the downregulation of miR-210 on the expressions of apoptosis-related proteins (Bax, caspase-3, and Bcl-2).

In MCF-7/DDP cells, miR-210 was downregulated by transfecting miR-210 inhibitor, and the changes in the expressions of apoptosis-related proteins (Bax, Bcl-2, and caspase-3) are examined by Western blotting. It was discovered that after miR-210 was knocked down, the expressions of Bax and caspase-3 were elevated, while the expression of Bcl-2 was decreased (Figure 4).

### 3.5. Effect of the miR-210 Downregulation on the JAK-STAT Signaling Pathway

**Figure 5:**
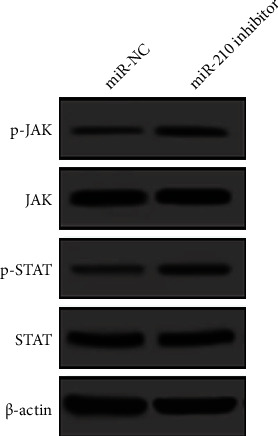
Changes in the expressions of the JAK-STAT signaling pathway-related proteins detected by Western blotting.

The expression of miR-210 was further downregulated in MCF-7/DDP cells by transfecting miR-210 inhibitor, and Western blotting was carried out to analyze the changes in the expressions of the JAK-STAT signaling pathway-related proteins (p-JAK, JAK, p-STAT, and STAT). The results (Figure 5) demonstrated that after knocking down miR-210, the expression levels of p-JAK and p-STAT were raised, but those of JAK and STAT did not change significantly, implying the that downregulation of miR-210 can activate the JAK-STAT signaling pathway.

### 3.6. Prediction and Verification of miR-210 Target Genes

**Figure 6:**
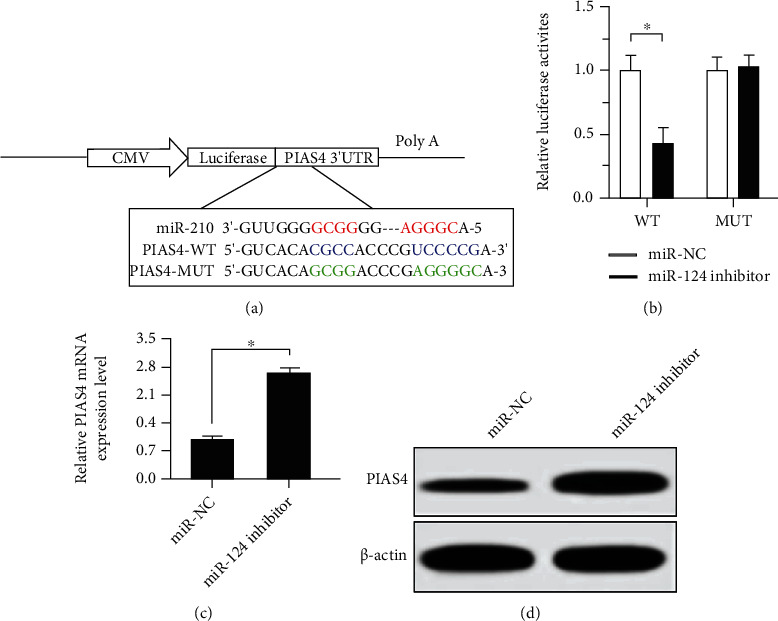
Verification of the miR-210 target gene. (a) The site matching the miR-210 sequence in the PIAS4 3′UTR is predicted using an online website. (b) Dual-luciferase reporter gene is used to detect the effect of miR-210 on the luciferase activity of PIAS4. (c, d) Changes in the mRNA and protein expression levels of PIAS4 examined via qPCR and Western blotting (^∗^*p* < 0.05).

Potential miR-210 target genes were predicted using the miRNA target gene database TargetScan, the results of which displayed that PIAS4 was a potential miR-210 target. PIAS is one of the regulatory factors of the JAK-STAT pathway [14]; so, it was taken as the preferred target gene. First, whether miR-210 can directly target PIAS4 was studied. MCF-7 cells were transfected with PIAS4 3′UTR reporter gene plasmid or Mut reporter gene plasmid and miR-NC or miR-124 mimics, and the luciferase activity was tested after 48 h. The results (Figure 6(b)) proved that the cotransfection with miR-210 and pmirGLO-PIAS4-WT remarkably inhibited the luciferase activity (*p* < 0.05), while the cotransfection with miR-210 and pmirGLO-PIAS4-Mut had no obvious impact on the luciferase activity. In order to further verify the targeting effect between miR-210 and PIAS4, miR-210 was knocked down in MCF-7/DDP cells, and changes in PIAS4 were observed at the mRNA and protein levels, respectively. The results (Figures 6(c) and 6(d)) confirmed that the downregulation of miR-210 evidently raised the mRNA and protein expression levels of PIAS4. In other words, the above results prove that miR-210 can directly target the 3′UTR of the PIAS4 gene to inhibit its expression.

## 4. Discussion

Breast cancer is one of the most common gynecologic malignant tumors, and chemotherapy still exerts a crucial effect in the treatment of breast cancer. However, the side effects of drugs seriously reduce the quality of life of patients. In addition, some tumor cells lack sensitivity to chemotherapeutic drugs or develop multidrug resistance, thus resulting in chemotherapy failure and tumor recurrence [15]. Therefore, studying new chemical sensitizing drugs with high efficiency and low toxicity will become an effective strategy to improve antitumor effect and delay recurrence.

Drug resistance of tumor cells is mostly acquired drug resistance, whose generation mechanism is very complex. A majority of studies have indicated that drug resistance involves the combined action of multiple mechanisms [16]. The upregulation of the expression of drug resistance-related proteins in tumor cells causes the efflux and uptake reduction of chemotherapeutic drugs, which further lead to a decrease in therapeutic concentration of drugs contained in cells, changes in drug action targets, and the abnormal expression of intracellular enzyme system, thereby resulting in the enhancement of nuclear DNA damage repair and cell detoxification function as well as the inhibition of tumor cell apoptosis due to the elevation of the expression level of antiapoptosis genes [17, 18]. MiRNAs are a type of highly conserved and short-length RNAs not encoded by proteins, and they play roles by negatively regulating the gene expression. Besides, miRNAs play a pivotal regulatory role in drug efficacy through regulating drug efflux, cell apoptosis, epithelial-mesenchymal transition (EMT), and tumor stem cells [19]. According to studies, the expressions of many miRNAs in breast cancer are increased or decreased, which contributes to the occurrence and development of the disease and affects drug sensitivity. For example, the overexpression of miR-451 makes breast cancer cells sensitive to doxorubicin [20], the elevation of miRNA-21 is related to the acquired trastuzumab resistance [21], and miR-106b-93-25 cluster leads to the activation of EMT and resistance to doxorubicin and paclitaxel [22]. However, the correlation between miR-210 and breast cancer chemosensitivity has not been clarified. Hence, this study is aimed at exploring the correlation between the miR-210 expression and chemoresistance of breast cancer MCF-7 cells. Mir-210 plays a role in various malignant diseases, such as ovarian, lung, and gastric cancer. Weijun et al. [23] showed that high expression of mir-210 could significantly inhibit the proliferation of ovarian cancer cells. Migration and invasion abilities serve as potential therapeutic targets for ovarian cancer. Wang et al. [24] showed that mir-210 can target to regulate the invasion and proliferation ability of lung cancer cells. Previous studies have found that the resistance of drug-resistant cells MCF-7/DDP to cisplatin was significantly higher than that of the control parent MCF-7, and the expression level of miR-210 in MCF-7/DDP was significantly higher than that of the control parent MCF-7. This suggests that the expression of miR-210 may be related to the chemotherapy resistance of breast cancer cells MCF-7. After that, miR-210 inhibitor was transfected to downregulate the miR-210 expression. MCF-7/DDP cells showed a significant decrease in resistance to DDP, indicating a significant increase in sensitivity to DDP. Moreover, the reduction of miR-210 also increased the apoptosis of MCF-7/DDP cells, which affected the apoptosis-related proteins and JAK-STAT signaling pathway-related proteins.

The JAK-STAT pathway is a very important cellular regulatory mechanism, which controls its activation and inactivation through many protein interactions, and exerts a variety of biological functions in normal cells and transformed cells. For a long time, people have considered it to be a potentially valuable target for drug development [25]. Yang et al. [26] researched that the activation of the JAK-STAT pathway can affect the sensitivity of lung cancer cells to targeted drugs and play a key role in the formation of lung cancer targeted therapy resistance. Guo [27] and other studies found that activation of the JAK-STAT pathway can downregulate the apoptosis sensitivity of liver cancer cells, leading to the emergence of liver cancer chemotherapy resistance. Interference with this pathway is obviously a promising treatment strategy, but it is necessary to correctly understand its core role in normal cell and tissue physiology and possible side effects related to its inhibition. Previous studies have shown that the expression of PIAS4 can activate the JAK-STAT pathway. The luciferase reporter gene detection in this experiment found that the expression of miR-210 can significantly reduce the activity of PIAS4 3′UTR wild type. However, there is almost no effect on the activity of the target site mutation PIAS4 3′UTR mutant, which proves that miR-210 can directly target the 3′UTR of the PIAS4 gene to inhibit its transcriptional activity. Therefore, miR-210 regulates that breast cancer cell MCF-7 chemotherapy resistance may be accomplished by targeting PIAS4 to regulate the JAK-STAT signal transduction pathway.

To sum up, our research shows that there is a correlation between the expression of miR-210 and the chemotherapy resistance of breast cancer cells MCF-7. By targeting PIAS4, it modulates the JAK-STAT signal transduction pathway and affects the sensitivity of breast cancer chemotherapy. In addition, it is conducive to the understanding of the role of miR-210 in breast cancer chemotherapy, which may provide a theoretical basis for breast cancer chemotherapy strategies.

## Data Availability

The datasets used and/or analyzed during the current study are available from the corresponding author on reasonable request.
